# PpAKR1A, a Novel Aldo-Keto Reductase from Physcomitrella Patens, Plays a Positive Role in Salt Stress

**DOI:** 10.3390/ijms20225723

**Published:** 2019-11-14

**Authors:** Lu Chen, Fang Bao, Shuxuan Tang, Enhui Zuo, Qiang Lv, Dongyang Zhang, Yong Hu, Xiaoqin Wang, Yikun He

**Affiliations:** 1 College of Life Sciences, Capital Normal University, Beijing 100048, China; 18811429649@163.com (L.C.); 5838@cnu.edu.cn (F.B.); 18801108621@163.com (S.T.); zeh216085@126.com (E.Z.); 13718531712@126.com (Q.L.); zdongy@163.com (D.Z.); hybrave@163.com (Y.H.); 2 Beijing Advanced Innovation Center for Tree Breeding by Molecular Design, Beijing University of Agriculture, Beijing 102206, China

**Keywords:** *Physcomitrella patens*, salt stress, methylglyoxal, aldo-keto reductase, reactive oxygen species, detoxification

## Abstract

The moss *Physcomitrella patens* is tolerant of highly saline environments. In plants, salinity stress may induce the production of toxic reactive carbonyl species (RCS) and oxidative damage. Aldo-keto reductases (AKRs) are a large group of NADP-dependent oxidoreductases involved in RCS detoxification. However, many members in this superfamily remain uncharacterized. In this study, we cloned and characterised a putative AKR1 from *P. patens*, named PpAKR1A. Notably, the transcription level of *PpAKR1A* was induced by salt and methylglyoxal (MG) stress, and the recombinant PpAKR1A protein catalysed the reduction of toxic aldehydes. *PpAKR1A* knockout mutants of *P. patens* (*ppakr1a*) were sensitive to NaCl and MG treatment, as indicated by much lower concentrations of chlorophyll and much higher concentrations of MG and H_2_O_2_ than those in WT plants. Meanwhile, *ppakr1a* plants exhibited decreases in the MG-reducing activity and reactive oxygen species-scavenging ability in response to salt stress, possibly due to decreases in the activities of antioxidant enzymes such as superoxide dismutase (SOD), catalase (CAT) and peroxidase (POD). Our results indicate that PpAKR1A is an aldo-keto reductase that detoxifies MG and thus plays an important role in salt stress tolerance in *P. patens*.

## 1. Introduction

Aldo-keto reductases (AKRs) are a superfamily of NADH-dependent oxidoreductases, including aldehyde reductase, aldose reductase and xylose reductase. AKRs are found in animals, plants and bacteria and have a wide variety of natural and foreign substrates [1]. For example, aldehyde reductase catalyses the reductions of various types of aldehydes and participates in the detoxification of reactive aldehydes [2]. In plants, AKRs play an important role in reductive detoxification under abiotic stress conditions [3].

Stress damage leads to the formation of large amounts of reactive oxygen species (ROS). Subsequently, ROS cause oxidative damage to proteins, DNA and lipids, leading to the production of toxic reactive carbonyl species (RCS) such as acrolein, malondialdehyde (MDA), methylglyoxal (MG), glyoxal and 3-deoxyglucosone [4]. Of these, MG was recently identified as a signalling molecule in plants [5]. The dual nature of MG depends on the intracellular concentration. Specifically, this RCS has deleterious effects at high concentrations but beneficial effects at low concentrations [6,7]. MG regulates many aspects of plant growth and physiology, including seed germination, root development and stress tolerance [8,9,10,11]. However, MG production increases rapidly in response to environmental stress, with detrimental effects such as the generation of ROS, formation of advanced glycation end products and inactivation of antioxidant systems [12,13].

In terms of adaptative strategies, plants have developed an array of defence mechanisms to combat RCS (such as MG) toxicity. For example, AKRs possess the capacity to detoxify RCS. In rice, the expression of *AKR4C15* mRNA increased in response to dehydration and oxidative stress, and the protein OsAKR4C15 exhibited the highest catalytic efficiency for the metabolism of MDA. Considerably lower concentrations of MG and MDA were observed in transgenic Arabidopsis plants engineered to overexpress *OsAKR4C15* than in WT plants under both normal and stress conditions [14]. In Arabidopsis, AKR4C8, AKR4C9, AKR4C10 and AKR4C11 mediated the detoxification of sugar-derived RCS in response to environmental stress [15,16]. Transgenic tobacco plants engineered to overexpress the heterologous rice protein OsAKR1 exhibited increased resistance to high temperatures and oxidative stress, which was mediated by increased AKR activity and reduced concentrations of MDA and MG [17]. Moreover, the transcription of AKR genes was also increased in *Medicago sativa*, *Vigna mungo*, *Jatropha curcas* L., tomato and chickpea during environmental stress, and the accumulation of these AKRs increased the abiotic stress tolerance of the plants via RCS scavenging and membrane damage reduction [18,19,20,21,22]. However, little is known about the functions of AKRs in nonvascular plants.

*Physcomitrella patens*, a model plant of bryophyte, exhibits the features of early land plants and is characterised by its high tolerance to drought, salinity, osmotic stress and other abiotic stresses [23,24,25]. *P. patens* has a relatively small genome and a relatively low level of redundant gene function [26,27]. Given these traits, *P. patens* is a remarkable system for evolutionary studies of plant gene function. In this study, we identified a novel AKR gene in *P. patens*, named *PpAKR1A* (XP_024403295.1), and determined the role of the gene product as a regulator of MG detoxification and salt stress tolerance. We confirmed the AKR activity of PpAKR1A and demonstrated that *PpAKR1A* deletion mutant protonemata displayed severe growth inhibition in response to MG and high salt. Our results demonstrated that the involvement of PpAKR1A in the detoxification of RCS and protection of antioxidant enzymes in *P. patens* under conditions of salt stress.

## 2. Results

### 2.1. Expression of PpAKR1A in Response to Salt Stress

**Figure 1:**
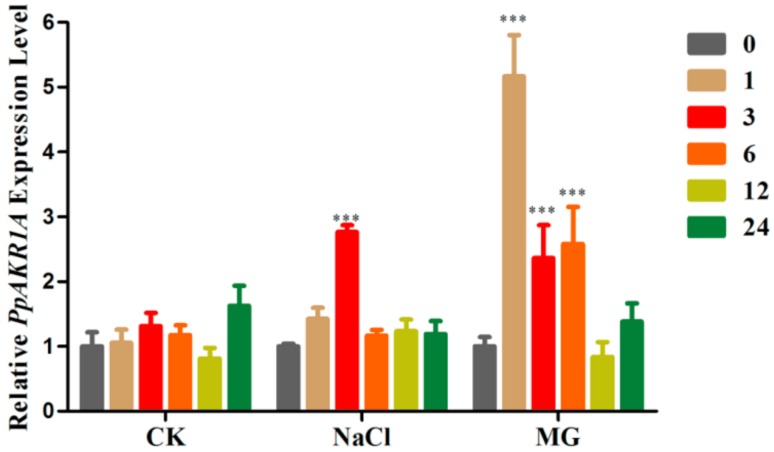
Expression pattern of *PpAKR1A* in wild type (WT) plants under untreatment (CK), 300 mM NaCl (NaCl) and 350 µM methylglyoxal (MG) treatment for 0, 1, 3, 6, 12 and 24 h. *Tublin* (XM_024520548.1) was used as a reference gene. Error bars indicate ± SD from three biological replicates (*** *p* ≤ 0.01).

Previous reports indicated that many AKRs were induced in plants during exposure to abiotic stresses [28,29,30,31]. To assess the potential role of *PpAKR1A* in abiotic stresses tolerance, we determined the effects of salt and MG stress on the expression pattern of this gene using qRT-PCR. However, we did not observe any changes in the abundance of *PpAKR1A* transcripts in the plants within 24 h after transfer to fresh BCDAT plates, irrespective of the stress type (Figure 1). However, when five-day-old protonemata were treated with NaCl, *PpAKR1A* transcription was induced rapidly from 0 to 3 h, followed by a rapid decrease nearly to the starting point at 6 h (Figure 1). In response to MG stress, *PpAKR1A* transcription was induced rapidly to more than fivefold the level observed within 1 h of treatment initiation (Figure 1). This result suggested that *PpAKR1A* is a response gene under saline conditions. 

### 2.2. Tissue Specificity and Subcellular Localisation of PpAKR1A

**Figure 2:**
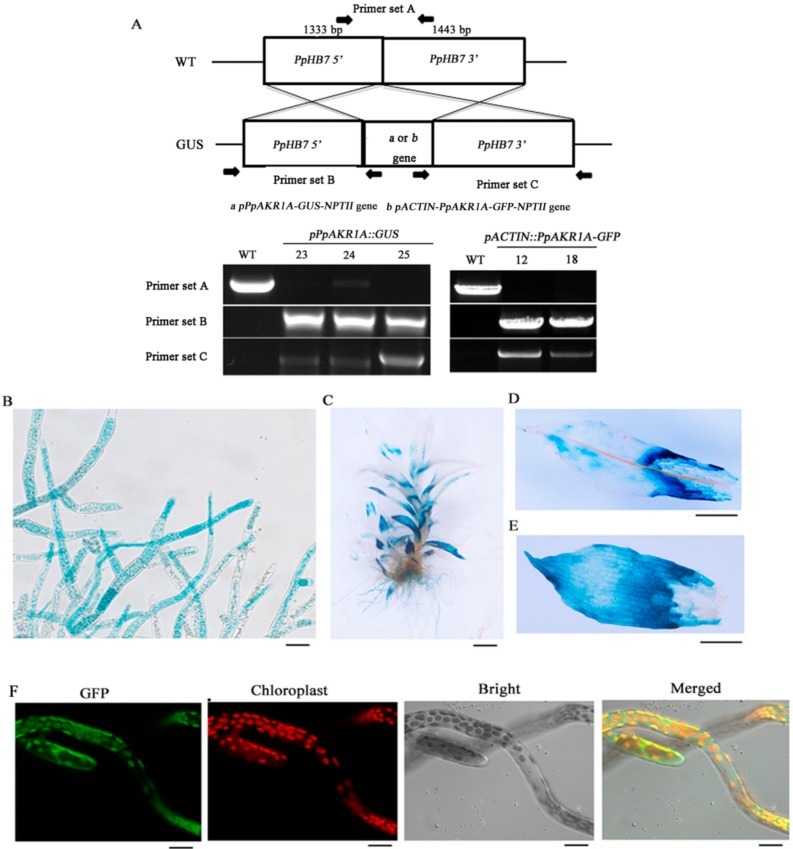
The tissue specificity of *PpAKR1A* gene and subcellular localization of PpAKR1A protein. Genomic PCR of the WT and *pPpAKR1A::GUS* or *pACTIN::PpAKR1A-GFP* transgenic lines was conducted using primers to amplify target sites (**A**) (i.e., *PpHB7* gene, *pPpAKR1A-GUS-NPTII* gene or *pACTIN-PpAKR1A-GFP-NPTII* gene). Tissue specificity of *pPpAKR1A::GUS* expression in protonema (**B**), gametophyte (**C**), phylloids at the top of gametophyte (**D**), and phylloids at the base of gametophyte (**E**). Subcellular localization of PpAKR1A::GFP fusing protein in the protonema (**F**). Scale bar = 20 µm (**B**,**C**,**F**) or 50 µm (**D**,**E**).

Next, an analysis of the *PpAKR1A* gene/promoter fusion GFP/GUS was conducted by introducing the *pACTIN::PpAKR1A-GFP-NPTII*, and *pPpAKR1A::GUS-NPTII* sequences into the WT genome via homologous recombination (Figure 2A). Consequently, we generated the transgenic plant strains *pACTIN::PpAKR1A-GFP* (Lines #12 and #18) and *pPpAKR1A::GUS* (Lines #23, #24 and #25). The different lines of *pPpAKR1A::GUS* and *pACTIN::PpAKR1A-GFP* all exhibited a similar phenotype when grown on BCD (gametophytes) and BCDA (protonemata) medium.

To characterise the pattern of *PpAKR1A* expression, transgenic *pPpAKR1A::GUS* plants were stained with GUS solution. GUS reporter expression was observed ubiquitously in both the protonema and gametophore tissues, with the strongest staining detected at the tip of the protonema and the bases of old gametophyte leaves (Figure 2B–E). In an analysis of the subcellular localisation of *PpAKR1A* in *pACTIN::PpAKR1A-GFP* transgenic plants, GFP fluorescence was detected mainly in the chloroplasts and cytosol of protonema cells (Figure 2F).

### 2.3. Response of PpAKR1A Knockout Mutants to Salt Stress

**Figure 3:**
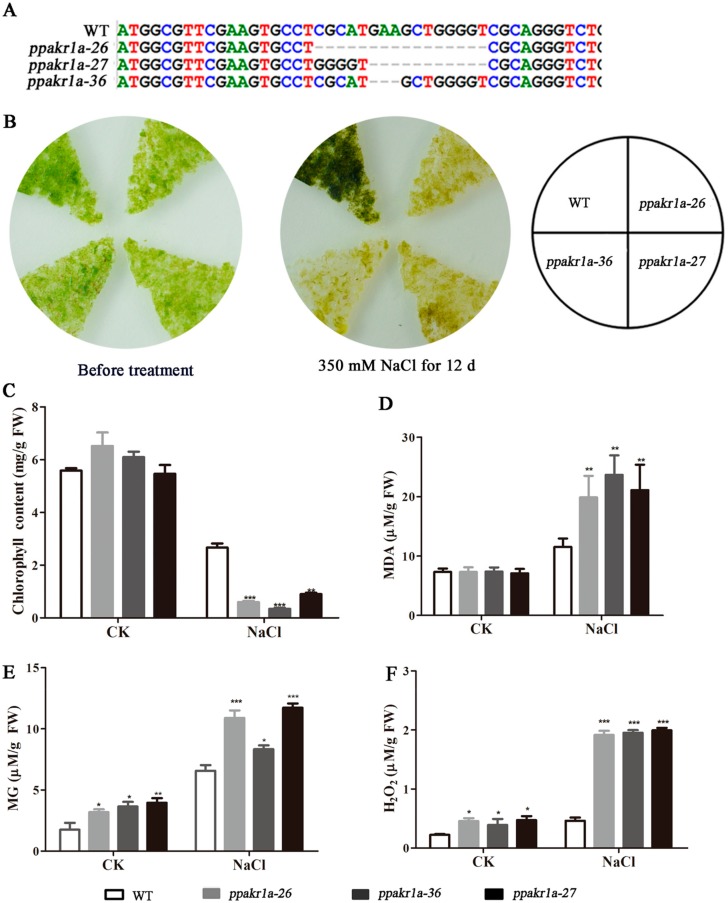
Phenotypic and physiological analysis of *ppakr1a* mutants under untreatment (CK) and salt stress (NaCl). Partial genomic sequences of WT and *ppakr1a* alleles (**A**). Representative photograph of *ppakr1a* and WT plants on BCD media with 350 mM NaCl (**B**). Total chlorophyll contents of WT and *ppakr1a* protonema after 350 mM NaCl treatment for 12 days (**C**). MG (**D**), MDA (**E**), and H_2_O_2_ (**F**) contents in protonema after 400 mM NaCl treatment for 5 days. Error bars indicate ±SD from three biological replicates. Significant differences were analyzed by *t*-test (* *p* ≤ 0.1, ** *p* ≤ 0.05, and *** *p* ≤ 0.01).

To acquire *PpAKR1A* transgenic plants, we transfected WT *P. patens* plants with *PpAKR1A* sgRNA-expressing plasmids. To obtain loss-of-function mutants, we used the CRISPR/Cas9-mediated method of gene editing [32]. Subsequently, we used PCR to identify three lines that harboured mutations involving a small insertion or deletion in the first exon of *PpAKR1A* (lines #26, #27 and #36; Figure 3A). Line #26 exhibited a deletion of 16 base pairs between nucleotides (nts) 19 and 34 after the translational initiation site. Line #27 exhibited a deletion from nt 24 to nt 34, C-G substitutions at nts 19 and 21 and an A-G substitution at nt 22. Line #36 exhibited deletions of nts 24 and 26. These three independent *ppakr1a* lines were used in further studies.

Previous reports demonstrated that the overexpression of AKR-encoding genes could promote salinity tolerance in different plant species [33], suggesting that these enzymes participate in salt-stress responses. Here, we compared the phenotypes of WT and *ppakr1a* plants in response to NaCl treatment. After treatment with 350 mM NaCl for 12 days, the protonemata of WT plants continued to grow and were greener in colour than before treatment. In comparison, *ppakr1a* protonemata exhibited severe growth suppression and a slightly browned colour (Figure 3B). Moreover, the total chlorophyll content, one of the most sensitive indicators of plant cellular damage, was approximately twofold greater in the WT protonemata than in the *ppakr1a* protonemata (Figure 3C).

AKRs contribute to salinity tolerance by detoxifying RCS [12,19,34]. Therefore, we measured the concentrations of MG and MDA in the mutant and WT plants under salt conditions. Lipid peroxidation is usually associated with increased free radical production [35]. We also examined the concentration of H_2_O_2_, a generator of oxygen free radicals [36]. We observed a small difference in the MDA concentrations between WT and *ppakr1a* protonemata under normal conditions. Although NaCl treatment enhanced the accumulation of MDA in both WT and *ppakr1a* protonemata, this accumulation was much greater in the latter group (Figure 3D). Unexpectedly, the H_2_O_2_ concentrations were approximately onefold higher in *ppakr1a* protonemata than in WT protonemata under normal growth condition, and this difference increased significantly during salt treatment to yield an approximately fourfold difference between the *ppakr1a* and WT plants (Figure 3F). Similarly, the *ppakr1a* plants exhibited increased MG accumulation (Figure 3E).

### 2.4. PpAKR1A Knockout Mutants Exhibit Reduced Tolerance to Methylglyoxal

**Figure 4:**
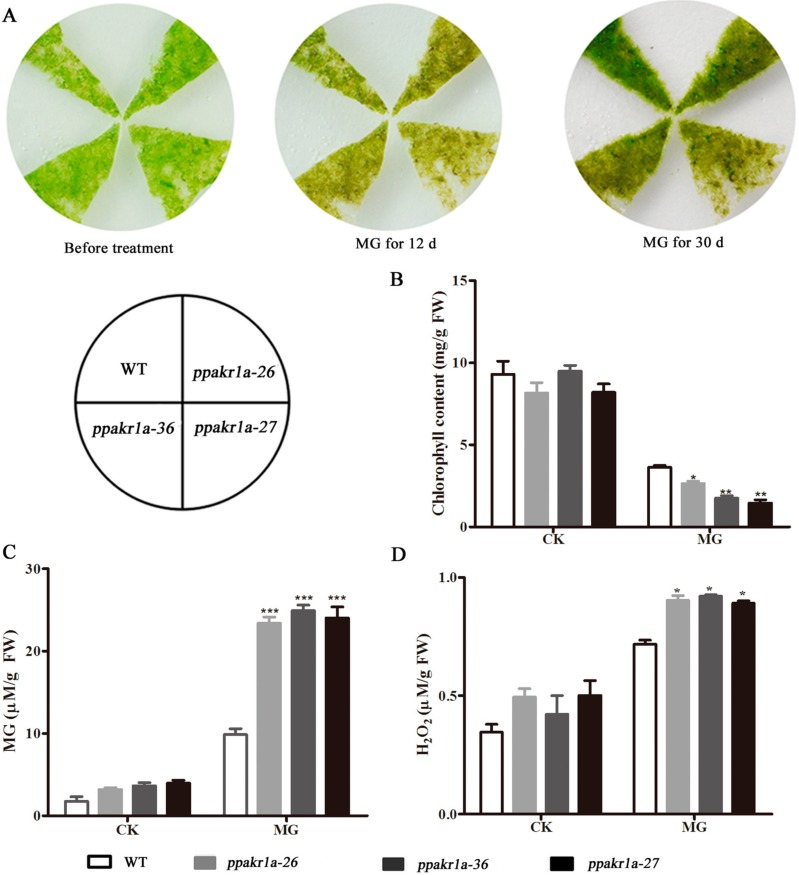
Phenotypic and physiological analysis of *ppakr1a* mutants under untreatment (CK) and methylglyoxal (MG) treatment. Representative photograph of *ppakr1a* and WT plants on BCD media with 350 µM MG (**A**). Total chlorophyll contents of WT and *ppakr1a* protonema after 30 days of MG treatment (**B**). MG (**C**) and H_2_O_2_ (**D**) contents in protonema after eight days of MG treatment. Error bars indicate ± SD from three biological replicates. Significant differences were analyzed by *t*-test (* *p* ≤ 0.1, ** *p* ≤ 0.05, and *** *p* ≤ 0.01).

As plant AKRs catalyse the breakdown of MG, we assumed that mutations in *PpAKR1A* might reduce the tolerance of plants to MG stress. Under normal conditions, we did not observe any obvious differences in growth between the three *ppakr1a* lines and the WT line. After treatment with 350 µM MG for 12 days, however, all the *ppakr1a* lines exhibited growth suppression (Figure 4A) and decreases in the total chlorophyll contents (Figure 4B). In addition, the MG level was approximately onefold higher in *ppakr1a* plants than in WT plants (Figure 4C), and a similar pattern was observed with respect to the H_2_O_2_ concentrations (Figure 4D).

### 2.5. Decreased MG Reducing Activity in PpAKR1A Knockout Mutants

**Figure 5:**
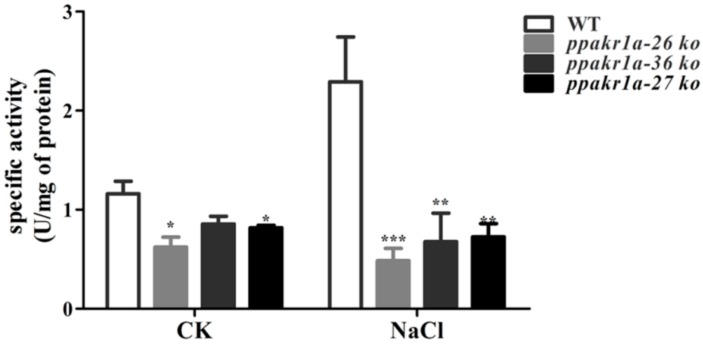
Activity of MG reduction in *ppakr1a* and WT plants under untreatment (CK) and 400 mM NaCl treatment (NaCl) for five days. Activity of MG reduction was measured on five-day-old protonemata extracts with 500 µM MG as a substrate. Error bars indicate ± SD from three biological replicates. Significant differences were analyzed by *t*-test (* *p* ≤ 0.1, and *** *p* ≤ 0.01).

Because of the high concentrations of MG observed in *ppakr1a* knockout mutants, we monitored the MG-reducing activities in the plants. Notably, we observed a lower MG reducing activity in *ppakr1a* protonemata than in WT protonemata under normal growth conditions. Under salt treatment, this difference increased significantly, such that the MG reducing activity was approximately twofold higher in WT plants than in *ppakr1a* plants (Figure 5). These results suggested that *ppakr1a* mutants decreased activity of MG reduction, thereby increasing the accumulation of MG in plants.

### 2.6. Decreased Activities of ROS-Scavenging Enzymes in PpAKR1A Knockout Mutants

**Figure 6:**
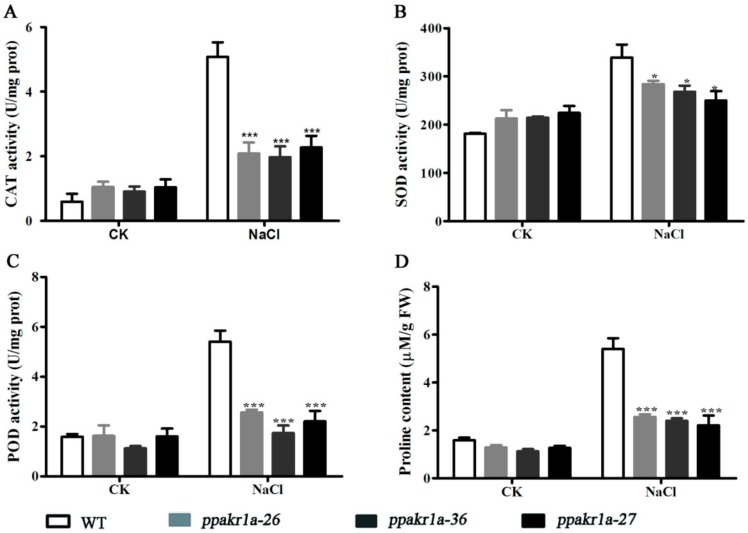
Activity of ROS scavenging enzymes and proline levels in *ppakr1a* and WT plants under untreatment (CK) and 400 mM NaCl treatment (NaCl) for 5 days. CAT (**A**), SOD (**B**), POD (**C**), and proline (**D**) levels. Error bars indicate ±S D from three biological replicates. Significant differences were analyzed by *t*-test (* *p* ≤ 0.1, and *** *p* ≤ 0.01).

Because of the high concentrations of H_2_O_2_ in *PpAKR1A* knockout mutants, We examined the activities various ROS-scavenging enzymes, such as superoxide dismutase (SOD), catalase (CAT) and peroxidase (POX). We also examined the proline concentration, as this is an important index of plant stress resistance [36,37]. Specifically, a higher concentration of proline is associated with stronger resistance to stress. We observed a significantly lower proline concentration and significantly reduced SOD, POD, and CAT activity concentrations in *ppakr1a* plants than in WT plants during NaCl treatment (Figure 6A–D). These results implied that *PpAKR1A* may be responsible for the maintenance of ROS-scavenging potential in cells, thereby improving the resistance of plants to environmental stress.

### 2.7. Bacterially Expressed Recombinant PpAKR1A Protein Effectively Reduced Toxic Aldehydes

The above results suggested that PpAKR1A might exhibit AKR activity, particularly the catalytic reduction of a wide range of aldehydes and sugars. Accordingly, we investigated the kinetics of the reduction of various aldehyde (MG, glyoxal, and acrolein) and sugar substrates (glucose and xylose) by recombinant PpAKR1A, using NADPH as a cofactor. We determined that PpAKR1A could reduce all tested aldehydes, but did not reduce the tested sugars. Of the aldehydes, PpAKR1A exhibited the highest catalytic efficiency with MG (Table 1).

## 3. Discussion

Soil salinity is a major environmental factor that affects plant growth and reduces crop yields worldwide [28,38]. Exposure to high salt (e.g., NaCl) conditions induces toxicity mediated by Na^+^ and Cl^−^ ions, oxidative stress, and disruption of the osmotic potential in plants, thereby reducing the absorption of water and nutrients from the soil. These changes reduce both the productivity and yields of farms [39,40]. To adapt to salt stress, plants have developed various mechanisms involving detoxifying enzymes.

Recent research suggests that *Pseudomonas PsAKR1* is responsible for salt tolerance in plants. During treatment with 150 or 300 mM NaCl, a transgenic tobacco strain engineered to overexpress *PsAKR1* exhibited low concentrations of Na^+^ ions and highly stable membranes in the root and shoot, resulting in increases in the root growth and biomass. Additionally, *PsAKR1*-overexpressing plants exhibited increased survival and decreased concentrations of MDA, H_2_O_2_ and MG in response to NaCl treatment [34]. However, few studies have explored the function of AKR1 in plants. In this study, we revealed the role of *PpAKR1A* and associated mechanism of regulation during salt stress in the moss species, *P. patens*.

### 3.1. PpAKR1A Enhances Salt Resistance in P. patens

In vascular plants, increased AKRs expression is accompanied by elevated stress tolerance [3,19]. Plant AKRs play an important role in oxidative damage induced by environmental stress [17,41]. In *P. patens*, we observed that *PpAKR1A* expression was rapidly induced by exposure to high salinity (Figure 1), suggested that this gene and its product might be involved in salt stress-tolerance in this moss species. To explore the role of *PpAKR1A* in salt stress, we generated *ppakr1a* mutant strains of *P. patens* using the CRISPR/Cas9 system, and observed that exposure to NaCl led to severe growth suppression and browning; in contrast, the WT plants remained robust (Figure 4A). 

The concentrations of total chlorophyll, MG, MDA and H_2_O_2_ are known as important physiological indicators of plant stress resistance [17,42,43,44]. Previous studies observed much higher concentrations of total chlorophyll and much lower concentrations of MG, MDA, and H_2_O_2_ in transgenic plants engineered to overexpress AKRs than in WT plants during salt stress [33,34]. These patterns indicate that AKRs are responsible for RCS detoxification in plants, and thereby improve resistance to salt stress. In our study, we also measured the concentrations of total chlorophyll, MG, MDA and H_2_O_2_ in *ppakr1a* and WT plants exposed to NaCl. As expected, the concentrations of total chlorophyll were much lower and the concentrations of MG, MDA, and H_2_O_2_ were much higher in *ppakr1a* plants than in WT plants under salt stress (Figure 4B–E). These findings further suggest that PpAKR1A plays an important role in RCS detoxification and resistance to salt stress in plants.

In plants, increased ROS-scavenging enzyme activity and decreased H_2_O_2_ concentrations are associated with improved tolerance to abiotic stresses [45,46,47]. SOD, CAT and POD are enzymes that scavenge ROS. Here, we have provided experimental evidence demonstrating much lower levels of SOD, CAT, and POD activity in *ppakr1a* plants than in WT plants under salt stress, leading to significant increases in the concentrations of H_2_O_2_ in *ppakr1a* plants (Figure 6). Additionally, we observed much lower concentrations of proline in *ppakr1a* plants than in WT plants during salt stress, suggesting that the former plants exhibited a decreased capacity for osmotic regulation. In summary, our results indicated that PpAKR1A plays a positive role in the response to salt stress. 

### 3.2. The Mechanism of PpAKR1A-Mediated Regulation in Response to Salt Stress

A phylogenetic analysis of plant AKR orthologues revealed that *PpAKR1A* is related closely to JcAKR in *Jatropha curcas L.* (Figure S1), a protein responsible for salt tolerance. Notably, bacterial and yeast cells that expressed *JcAKR* were more tolerant than controls when exposed to 200 mM NaCl. Both the expression of *JcAKR* and enzyme activity of JcAKR increased in response to salt stress. Moreover, in *J. curcas*, JcAKR plays a crucial role in MG detoxification [19]. MG, a cytotoxic α,β-dicarbonyl compound, is produced by plants via sugar and amino acid metabolism and acts as a signal molecule during responses to stress [48,49,50]. In Arabidopsis, all four AKR4C(s) can metabolise MG [14,15]. To study the mechanism of PpAKR1A regulation under salt stress, we analysed the enzyme activity of PpAKR1A and the concentrations of MG and confirmed that PpAKR1A catalysed the reduction of MG (Table 1). As expected, the concentration of MG was much higher in *ppakr1a* plants than in of WT plants, regardless of whether the plants were exposed to NaCl (Figure 4D). In addition, *ppakr1a* plants contained higher concentrations of MG than WT plants after exposure to MG. Similar to NaCl, MG exposure led to a reduced level of chlorophyll and increased level of H_2_O_2_ in *ppakr1a* plants relative to WT plants (Figure 5). This pattern might be attributable to the poor availability of PpAKR1A enzyme for detoxification, which would have led to cytotoxicity and plant growth suppression. Early studies showed that exogenous MG could inhibit plant growth and development [51]. We observed that *PpAKR1A* mutants exhibited a weaker phenotype in response to MG treatment vs. NaCl treatment, and this discrepancy might be attributable to other regulatory mechanisms associated with salt stress tolerance. Our results suggested that MG detoxification during salt stress is major function of PpAKR1A in *P. Patens*.

RCS are known to affect the accumulation of ROS in plants, predominantly via the formation of protein carbonyls that affect the functions of ROS scavenging enzymes [12]. The increased concentrations of H_2_O_2_ in *ppakr1a* mutants might be attributable to RCS-mediated decreases in the activities of SOD, CAT and POD. Our results were similar to those of a previous study in which AKR appeared to mediate salt tolerance by detoxifying RCS and thus maintaining ROS homeostasis [34]. In other words, *PpAKR1A* may decrease the level of RCS, which could in turn help to maintain the activity of ROS scavenging enzymes to reduce the level of ROS. Therefore, PpAKR1A could enhance tolerance to oxidative damage during salt stress in *P. Patens.*

## 4. Materials and Methods

### 4.1. Plant Materials and Stress Treatments

*P. patens* (Hedwig) ecotype ‘Gransden 2004’ was used as the WT strain. Protonema tissues were grown axenically on BCDA agar medium containing 0.5% (*w*/*v*) glucose and 1 mM CaCl_2_ under a light intensity of 50 µmol m^−2^ s^−1^, a temperature of 25 °C and a day/night cycle of 16 h/8 h [26,52]. Five-day-old protonemata were transferred onto ammonium tartrate-free BCD medium supplemented with 350 mM NaCl (Sigma-Aldrich, St. Louis, MO, USA) or 350 µM MG (Sigma-Aldrich, St. Louis, MO, USA) for phenotypic analysis. To determinate the *PpAKR1A* transcript level under salt stress, five-day-old protonemata were grown on BCD medium containing 300 mM NaCl or 350 µM MG for 0, 1, 3, 6, 12 and 24 h. For studies of physiological parameters, five-day-old protonemata were grown on BCD medium with 400 mM NaCl or 350 µM MG for 5 or 8 days.

### 4.2. Quantitative Real-Time Reverse Transcription PCR (qRT-PCR) Analysis

Total RNA was extracted from plant tissues using the RNeasy Plant Mini Kit (Qiagen, Valencia, CA, USA). Single-stranded cDNA was synthesised from RNA using the PrimeScript RT reagent Kit with gDNA Eraser (Takara Bio, Dalian, China) according to the manufacturer’s instructions. The *P. patens* gene encoding tubulin (forward: GAGTTCACGGAAGCGGAGAG; reverse: TCCTCCAGATCCTCCTCATA) was used as a standard to normalise the cDNA expression concentrations. The qRT-PCR reactions were performed on an ABI7500 thermocycler (Applied Biosystems, Foster City, CA, USA) using the SYBR^®^ Premix ExTaqTM Kit (Takara Bio, Dalian, China) with primers specific for *PpAKR1A* (forward: GCTGGACGTGGACTATATCGATCT; reverse: CGCCGCCACAGTAATTTCG). The data were analysed using the 2^–∆∆CT^ method, and the reported relative gene-expression data represent the averages of three biological repeats. 

### 4.3. Plasmid Construction

To obtain gene knockout mutations using the CRISPR/Cas9 system, we subjected the first exon of *PpAKR1A* to a search of CRISPR RNA (crRNA: GAAGTGCCTCGCATGAAGCT) preceded by a PAM motif from *Streptococcus pyogenes* Cas9 (NGG or NAG). This search was conducted by applying the webtool CRISPOR V1 to the *P. patens* genome Phytozome V9 (http://crispor.tefor.net/crispor.py) [32]. The *PpAKR1A* sgRNA comprised a fusion of 20 bp of crRNA to 83 bp of the *S. pyogenes* tracrRNA scaffold (Fabien Nogué) under the control of the *P. patens* U3 or U6 promoter. A chemically synthesised fragment of *PpAKR1A* sgRNA (Synbio Technologies Institute, Suzhou, China) was subcloned into the pUC57 (GenScript) backbone using the Syno assembly mix (Synbio, Suzhou, China). Next, the PEG-mediated transformation protocol was adapted to deliver the *PpAKR1A* sgRNA plasmid together with a plasmid containing a human codon-optimised form of *S. pyogenes* Cas9 (Sp-hCas9) and pBNRF (Fabien Nogué) into *P. patens* protoplasts.

To construct the plasmid encoding the *PpAKR1A::GFP* fusion gene, the *PpAKR1A*-coding sequence (CDS) was amplified from WT cDNA (forward: ATGGCGTTCGAAGTGCCT; reverse: TCAGATTGTATGAAGGCCCTCT) inserted into the *AscI* and *StuI* sites of the pTFH15.3 vector (Mitsuyasu Hasebe) under the rice *Actin2* promoter to yield *pTFH15.3-Actin pro::PpAKR1A*. The GFP fragment from the pCAMBIA1302 vector (Invitrogen) was subsequently inserted into the *ApaI* site of *pTFH15.3-Actin pro::PpAKR1A.*

To conduct the *PpAKR1A promoter::GUS* reporter assay, a 1.5-kb fragment of the *PpAKR1A* promoter was amplified from WT genomic DNA by PCR using specific primers (forward: AGATTGCACTCATGTGTTCTCC; reverse: TGCGACACTTCCAGACCC). This amplified fragment was then digested with *SpeI* and *AscI* and cloned into the corresponding restriction sites of the pTFH15.3-GUS vector (Suxia Cui) to generate *pTFH15.3-PpAKR1A pro::GUS*. 

### 4.4. Transformation of P. patens

Transformation was performed using the standard PEG-mediated method [32,53]. To select transformants with CRISPR/Cas9-based mutations, fresh *P. patens* protoplasts were grown on BCDAT medium for 7 days and then transferred onto BCDAT selection medium containing 50 mg/L^−1^ G418. The surviving protoplasts-turned-colonies were then cultivated on G418-free BCD medium until they could be harvested. *PpAKR1A* was then amplified using specific primers (forward: GGAAATCCTGTAGTGAAACCCC; reverse: GAAGTATCCAAAAACGTCACACCT). The DNA amplification fragment was then sequenced to identify CRISPR-mediated indels. To select fusion GFP and GUS transgenic lines, fresh protoplasts were cultivated on BCDAT medium. After a 7-day regeneration period, the tissues were transferred to BCDAT selection medium supplemented with 25 mg/L G418. The integration of exogenous DNA into the genomic DNA of stable transformants was then screened via genomic PCR analyses with primer sets A (forward: GGAAATCCTGTAGTGAAACCCC; reverse: CGTCTATTCTGGAACCACACCGC), B (forward: CATCCTACAGTTTGGCAGTCCT; reverse: CGAGGTATGTAGGCGGTGC) and C (forward: CTAAACCTGGAGCCCAGACG; reverse: CTGCTGTAAGGACCGCAAGTAG).

### 4.5. Histochemical Analysis of GUS Activity

We performed a histochemical analysis of the *PpAKR1A*-promoter-driven GUS (*pPpAKR1A::GUS*) transgenic lines to detect β-glucuronidase (GUS) activity according to the manufacturer’s protocols [54]. Five-day-old protonema and 21-day-old gametophyte tissues were incubated in GUS staining solution (Sbjbio, Nanjing, China) at 37 °C for 10–12 h, in water for 10–60 min and in 70% ethanol for decolourisation. Finally, the protonema and gametophyte were analysed using a Zeiss Axio Imager M2 and Axio Zoom V16 M2 microscope (Stuttgart, Germany), respectively. The data represent three independent lines with similar staining patterns.

### 4.6. PpAKR1A–GFP Subcellular Localisation Analysis

The *pACTIN::PpAKR1A-GFP* transgenic lines were subjected to a GFP fluorescence analysis to determine the subcellular localisation of according to the manufacturer’s protocols [55]. Five-day-old protonemata were subjected to confocal imaging using a Zeiss LSM 780 microscope (Stuttgart, Germany). The data represent two independent lines that displayed similar GFP subcellular localisation patterns.

### 4.7. Physiological Parameter Measurements

The concentrations of total chlorophyll, malonyldialdehyde (MDA), proline, H_2_O_2_ and methylglyoxal (MG) were measured in 0.5 g samples of protonema tissue, as previously described [42,48,56,57,58]. The absorbance values were determined using a Beckman DU-640 spectrophotometer. The data analysis included more than three biological replicates per sample. 

### 4.8. Oxidative Enzyme Assays

The ROS-scavenging activities of SOD, CAT and POD in 0.5 g samples of protonema tissues were measured using enzyme-linked immunosorbent assays (ELISA) according to the manufacturer’s instructions (Jiancheng Bioengineering Institute, Nanjing, China).

### 4.9. Cloning and Expression of Recombinant Protein of PpAKR1A

Recombinant PpAKR1A protein was cloned and expressed in *E. coli* according to a previously described method [16]. Briefly, the coding region of *PpAKR1A* was amplified by RT-PCR using specific primers designed to introduce *BamHI* and *SalI* restriction sites (forward: CGCGGATCCATGGCGTTCGAAGTGCCT; reverse: ACGCGTCGACTCAGATTGTATGAAGGCCCTCT). The PCR product was then cloned into the corresponding restriction sites of the pMAL-c1expression vector (New England Biolabs (Beijing) Ltd., Beijing, China), and the resulting construct was transformed into *E. coli* BL21 cells (Novagen, Beijing, China). The transformed BL21 cells were cultured in Luria-Bertani (LB) medium containing 100 g/mL ampicillin at 37 °C until the absorbance at 600 nm reached approximately 1.0. Recombinant protein was induced by adding 1 mM isopropyl β-D-1-thiogalactopyranoside (IPTG )to the cultures of BL21 cells in LB medium for 30 min at 37 °C, after which the cells were cultured at 16 °C for 16 h in an orbital shaker at a speed of 220 rpm.

### 4.10. Purification Protein of the His-Fusion PpAKR1A

The His-fusion PpAKR1A was purified and analysed according to the manufacturer’s instructions [16,59], with a slight modification. The cells were collected and disrupted in lysis buffer (20 mM Tris, 150 mM NaCl, 1% Triton X-100 and 0.1 mM phenylmethanesulfonyl fluoride (PMSF), pH 7.0) by sonication. After centrifugation at 14,000 rpm and 4 °C for 15 min, the supernatant was mixed with Ni-NTA Agarose (Qiagen, Valencia, CA, USA), and the mixture was shaken gently for 12 h at 4 °C. After washing the mixture with lysis buffer to remove unbound protein, the bound protein was eluted using lysis buffer supplemented with 300 mM imidazole. The protein concentration was estimated using a Coomassie Blue binding method (Bio-Rad, Hercules, CA, USA), with bovine serum albumin as the standard. The purified protein was monitored by 12% sodium dodecylsulphate polyacrylamide gel electrophoresis (SDS–PAGE).

### 4.11. Enzyme Activity and Enzyme Kinetics

In vitro spectrophotometric enzyme assays were conducted in a 200-µL reaction volume containing 50 mM Tris-HCl buffer (pH 7.0), 150 µM NADPH, recombinant enzyme and substrate according to a previously described method [16].

The AKR activity in extracts of 1 g protonema tissue was measured as previously described [17]. The reaction mixture included the buffer and NADPH concentration as described above for the enzyme assays, as well as 500 µM MG as the substrate. The decrease in absorbance at 340 nm was measured in reactions containing purified protein and protonemata extracts for 2 min using a Beckman DU-640 spectrophotometer at 25 °C. The specific activity was calculated using the extinction coefficient of 6220 M^−1^ cm^−1^. The enzyme kinetic parameters were calculated using a non-linear regression of Michaelis–Menten data. The calculations were performed using GraphPad Prism, version 5.04.

## 5. Conclusions

**Figure 7:**
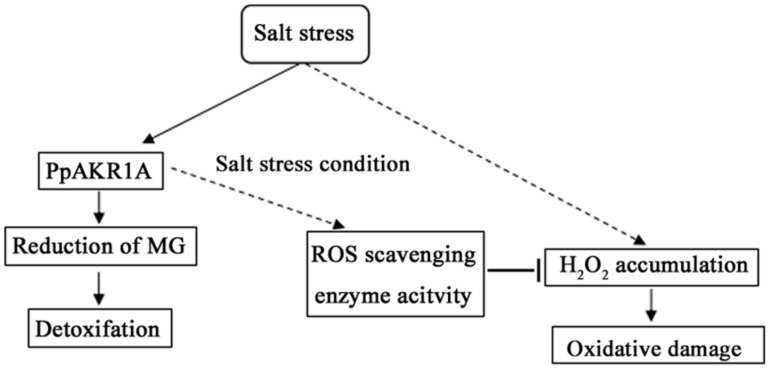
A proposed model for the role of PpAKR1A in regulating salt stress tolerance. Salt induces H_2_O_2_ accumulation, which causes oxidative damage to the cell. In response to salt stress, the transcription level of *PpAKR1A* was induced. Then, PpAKR1A protein could detoxify MG by catalyzing the reduction of MG and indirectly enhance ROS-scavenging enzymes activity like SOD, POD and CAT, which in turn inhibits H_2_O_2_ accumulation. Ultimately, plants are more tolerant to salt and oxidative stress.

PpAKR1A promotes salt stress tolerance in the moss *P. patens* by reducing MG (Figure 7). The *ppakr1a* mutant exhibited lower tolerance to salt stress, which was accompanied by the increased accumulation of MG and MDA and decreased concentrations of chlorophyll. The observed reduction in stress tolerance *ppakr1a* plants was attributable to RCS modifications, which would decrease the ROS scavenging capability and thus enhance oxidative stress indirectly. Our results demonstrated that PpAKR1A can promote salt-stress tolerance via the efficient detoxification of MG. Therefore, *PpAKR1A* may be a candidate gene in genetic engineering studies to increase agricultural production in salt-heavy soils.

## Figures and Tables

**Table 1:** Apparent kinetic constants of recombinant PpAKR1A.

Substrate	*K_m_* (mM)	*K_cat_* (min^−1^)	*k_cat_/K_m_* (min^−1^ mM^−1^)
*Aldehydes*			
Methylglyoxal	0.2262 ± 0.063	51.6 ± 3.3	228.6
Glyoxal	1.526 ± 0.237	3.78 ± 2.46	3.78
Acrolein	6.2 ± 1.114	33.42 ± 1.86	5.34
*Sugars*			
Glucose	nd	nd	nd
Xylose	nd	nd	nd

nd—not detected. All measurements were performed in three replicates.

## Supplementary Materials

Supplementary materials can be found at https://www.mdpi.com/1422-0067/20/22/5723/s1.

## Abbreviations

ROS	Reactive oxygen species
RCS	Reactive carbonyl
MG	Methylglyoxal
MDA	Malondialdehyde
AKR	aldo-keto reductases
qRT-PCR	real-time polymerase chain reaction
PCR	polymerase chain reaction
WT	wild type
CRISPR/Cas9	clustered regularly interspaced short palindromic repeats/CRISPR-associated protein 9
CAT	catalase
SOD	superoxide dismutase
POD	peroxidase
IPTG	isopropyl β-D-1-thiogalactopyranoside
PMSF	phenylmethanesulfonyl fluoride
SDS-PAGE	sodium dodecylsulphate polyacrylamide gel electrophoresis
LB	Luria-Bertani
PpAKR1A	a putative AKR1 from *P. patens*
