# Supplementary material for: PpAKR1A, a Novel Aldo-Keto Reductase from Physcomitrella Patens, Plays a Positive Role in Salt Stress
Source: Int J Mol Sci. 2019 Nov 14;20(22):5723. doi: 10.3390/ijms20225723 (PMC6888457; doi:10.3390/ijms20225723)
Supplement: Supplementary file 1 [file ijms-20-05723-s001.pdf]

Supplementary Materials

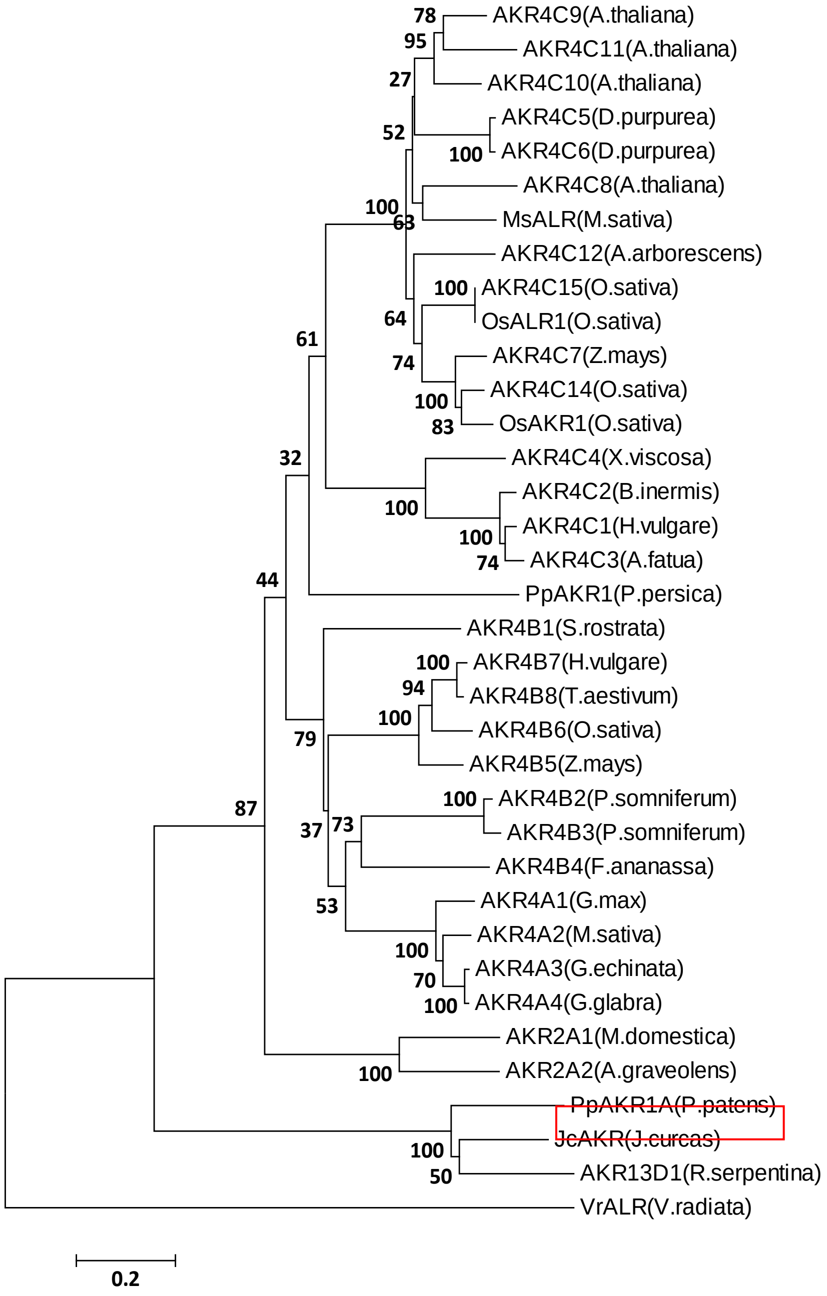

Figure S1. Phylogenetic analysis: PpAKR1A and its relationship with other published plant AKRs. MEGA5 was used for the construction of the tree using neighbour joining methodand clustal W program, with boot strap method taking 1000 replicates. The branch numbers refer to the bootstrap confidence. The accession numbers of PpAKR1A, JcAKR, PpAKR1, MsALR and VrALR are XP_024403295.1, KU513391, AB183148, CAA66205 and AAD53967.1 respectively, whereas the accession codes

for the other AKRs used in the construction of phylogenetic tree are available at http://www.med.upenn.edu/akr (Hyndman et al., 2003).

Sequence and biochemical properties of His-MBP-tagged-PpAKR1A

>protein

MAFEVPRMKLGSQGLEVSQQGLGCMGMSCFYGLPAPEQEMIDLIHYAVERG
VTFLDTSDMYGPHTNEVLVGKAIKGIRDKVQLATKFGNIFDQKGNVMVRGDP
EYVRQACEASLKRLDVDYIDLYYQHRLDKKVPIEITVAAMAELVKEKKVKYL
GLSEANASEIRRAHAVHPITAVQLEWSLWVRDLEKEIVPTCRELGISIVSYSPLG
RGFFAGYNPQEAKEGDFRKMYGRLSGENLAKNEKLRQRVMEIAEGKKCSIN
QLALAWVHHKGKDVVPIPGTTKKKNLDSNIQALQVTLTSEEMAELEAAVPEE
EVAGDRYGKATLQATWRYASTPPLSSWNPSSVTAEEGLHTI**

Number of amino acids: 354

Molecular weight: 39502.31

Theoretical pI: 6.29

Graph of kinetic constant of PpAKR1A

Methylglyoxal : Km=0.2262±0.06332, kcat = 51.7±3.317 min^{-1},

kcat/ Km = 228.7 $mM^{-1}min^{-1}$ $r^2 = 0.8653$

Substrate concentration range: 0.16-1.944 mM

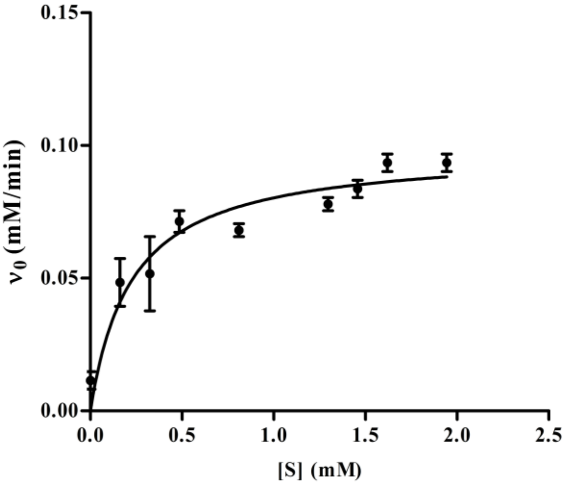

Figure S2. Graph of kinetic constant of PpAKR1A

Glyoxal: Km = 1.526±0.2378, kcat = 0.063±0.04113 s^{-1},

kcat/ Km =0.063 $s^{-1}mM^{-1}$ r^2 = 0.9708

Substrate concentration range: 0.3-8.5 mM

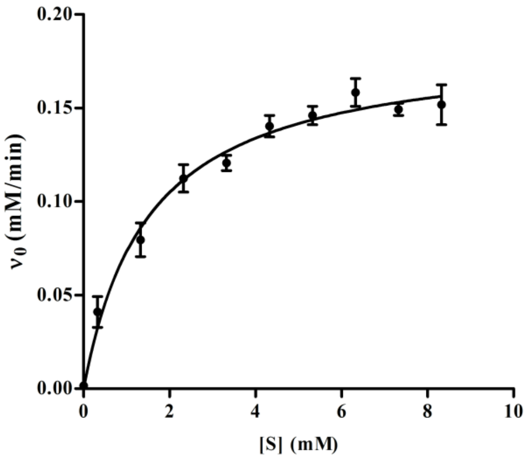

Figure S3. Graph of kinetic constant of PpAKR1A

Acrolein: Km = 6.2±1.114,   kcat =   0.557±0.0312 s^{-1},

kcat/ Km =0.089 $s^{-1}mM^{-1}$ r^2 = 0.9533

Substrate concentration range: 2.0-30 mM

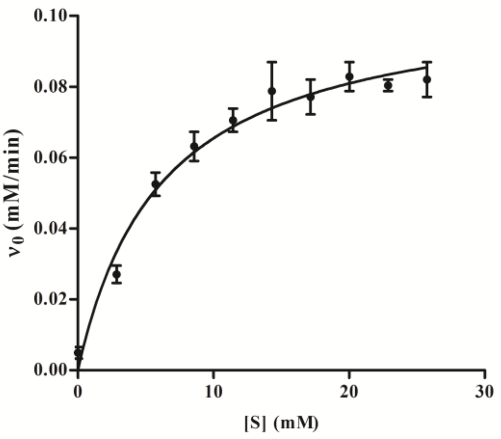

Figure S4. Graph of kinetic constant of PpAKR1A
